# Inhibitors of Foot and Mouth Disease Virus Targeting a Novel Pocket of the RNA-Dependent RNA Polymerase

**DOI:** 10.1371/journal.pone.0015049

**Published:** 2010-12-21

**Authors:** Ryan C. Durk, Kamalendra Singh, Ceili A. Cornelison, Devendra K. Rai, Kayla B. Matzek, Maxwell D. Leslie, Elizabeth Schafer, Bruno Marchand, Adeyemi Adedeji, Eleftherios Michailidis, Christopher A. Dorst, Jennifer Moran, Christie Pautler, Luis L. Rodriguez, Mark A. McIntosh, Elizabeth Rieder, Stefan G. Sarafianos

**Affiliations:** 1 Christopher Bond Life Sciences Center, Department of Molecular Microbiology and Immunology, University of Missouri School of Medicine, Columbia, Missouri, United States of America; 2 Department of Molecular Microbiology and Immunology, University of Missouri School of Medicine, Columbia, Missouri, United States of America; 3 Foreign Animal Disease Research Unit, United States Department of Agriculture, Agricultural Research Service, Plum Island Animal Disease Center, Greenport, New York, United States of America; Nanyang Technological University, Singapore

## Abstract

**Background:**

Foot-and-Mouth Disease Virus (FMDV) is a picornavirus that infects cloven-hoofed animals and leads to severe losses in livestock production. In the case of an FMD outbreak, emergency vaccination requires at least 7 days to trigger an effective immune response. There are currently no approved inhibitors for the treatment or prevention of FMDV infections.

**Methodology/Principal Findings:**

Using a luciferase-based assay we screened a library of compounds and identified seven novel inhibitors of 3Dpol, the RNA-dependent RNA polymerase of FMDV. The compounds inhibited specifically 3Dpol (IC_50_s from 2-17 µM) and not other viral or bacterial polymerases. Enzyme kinetic studies on the inhibition mechanism by compounds 5D9 and 7F8 showed that they are non-competitive inhibitors with respect to NTP and nucleic acid substrates. Molecular modeling and docking studies into the 3Dpol structure revealed an inhibitor binding pocket proximal to, but distinct from the 3Dpol catalytic site. Residues surrounding this pocket are conserved among all 60 FMDV subtypes. Site directed mutagenesis of two residues located at either side of the pocket caused distinct resistance to the compounds, demonstrating that they indeed bind at this site. Several compounds inhibited viral replication with 5D9 suppressing virus production in FMDV-infected cells with EC_50_ = 12 µM and EC_90_ = 20 µM).

**Significance:**

We identified several non-competitive inhibitors of FMDV 3Dpol that target a novel binding pocket, which can be used for future structure-based drug design studies. Such studies can lead to the discovery of even more potent antivirals that could provide alternative or supplementary options to contain future outbreaks of FMD.

## Introduction

The Foot-and-Mouth Disease Virus (FMDV) is a member of the *Aphthovirus* genus in the Picornaviridae family. There are seven known serotypes of FMDV: A, O, C, Asia 1, and Southern African Territories (SAT) 1, 2 and 3 [1]. Within these serotypes, over 60 subtypes have also been reported. Because of this diversity there is no universal vaccine, thus presenting challenges in the selection of vaccine strains [2]. The most effective FMD vaccines consist of chemically inactivated FMDV and can only offer complete protection after seven days of vaccination because of the time needed to trigger an immune response [3]. It has been proposed that a combination of vaccine and antivirals can be a more efficacious strategy to treat FMD-infected animals, contain the spreading of the disease, and reduce the number of animals that need to be slaughtered during outbreaks [3]. However, there are currently no approved anti-FMDV drugs for the treatment or prevention of FMD [4].

The FMDV genome is an 8.5kb uncapped, single-stranded RNA. It is translated as a single polyprotein, which in turn is cleaved into structural and non-structural proteins [5]. The non-structural protein that carries out RNA synthesis during transcription and replication is an RNA-dependent RNA polymerase (RdRp or 3Dpol). Because of their pivotal roles in the viral life cycle, viral polymerases have been a primary target for the development of antiviral agents. In fact, there are nearly 35 approved antiviral drugs that target polymerases of various pathogens [6], [7] [8], [9], [10], [11] [12]. Thus, 3Dpol of FMDV is an attractive target for chemotherapeutic intervention.

Among the compounds that target FMDV 3Dpol is ribavirin, a mutagenic nucleoside analogue, known to exhibit antiviral activity against a broad range of both DNA and RNA viruses [13], [14], [15], [16], [17], [18]. Suppression of FMDV replication in cell cultures requires relatively high concentrations of ribavirin (EC_50_ = 970 µM). In addition, a resistant mutation in 3Dpol (M296I) has been shown to decrease FMDV susceptibility to ribavirin [19], [20]. Recently, another compound, 2′-*C*-methylcytidine has been shown to inhibit FMDV at low µM concentrations, most likely through inhibition of 3Dpol [21]. Similarly, the pyrazinecarboxamide derivative T1106 [22] has been shown to be effective against FMDV. T1106 is converted to a triphosphate form by host enzymes and likely inhibits the replicase activity of FMDV 3Dpol, although the exact mechanism of FMDV inhibition by T1106 is unknown.

Here we report the discovery of seven novel specific inhibitors of FMDV 3Dpol, through screening of a chemical library of drug-like compounds. We show that at least one of the compounds has the ability to efficiently suppress viral replication without significant cytotoxicity. Finally, we also identified the inhibitor binding site, opening the way for structure-based drug design studies that should help discover highly potent inhibitors of FMDV.

## Results

### Development of 96-well plate screening assay for the detection of FMDV 3Dpol inhibitors

The principle of the method used in this study has been described previously [23] and is shown in Scheme 1. Briefly, the PP_i_ released from the polymerase reaction is converted to ATP in a reaction that uses adenosine 5′-phosphosulfate and is catalyzed by ATP sulfurylase. The ATP product provides the energy for the luciferase-catalyzed conversion of D-luciferin to oxyluciferin, with a concomitant release of photons. The sequence of reactions and scheme is as follows:

### Scheme 1



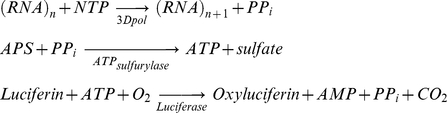



**Figure 1:**
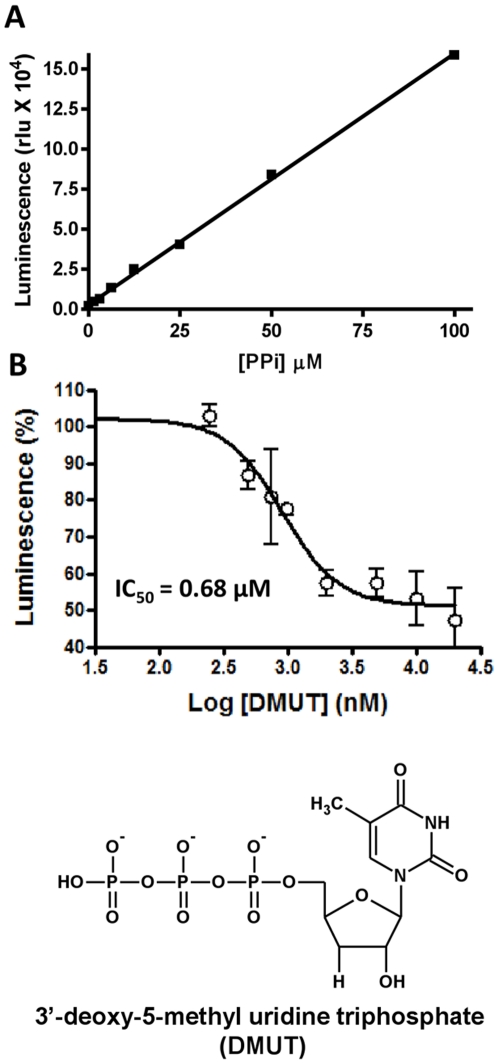
Standardization of luciferase-based RNA synthesis assay and validation using the DMUT inhibitor. (A) Light production as a function of exogenously added pyrophosphate (PPi). The amount of light generated by the coupled ATP sulfurylase and luciferase reactions is directly proportional to the amount of PPi added over a range of at least 2 logs (1–100 µM PPi). (B) Assay validation using 3′-deoxy 5-methyl-uridine-5′triphosphate (DMUT) as an inhibitor of FMDV 3Dpol (DMUT lacks a 3′OH required for RNA synthesis). Varying concentrations of DMUT incubated with 1.7 µM FMDV 3Dpol, 40 nM poly-rA/5′-Cy3-dT_18_, and 10 µM UTP at 37 °C for one hour prior to the addition of the ATP sulfurylase and luciferase assay components (n = 3, error bars are standard deviation from the mean).

We optimized the conditions for the light-generating reaction as described in the ‘Materials and Methods’ section. Moreover, we improved the cost-efficiency of the overall assay by decreasing the reagents' concentrations of the secondary (light-producing) reaction, while ensuring that they do not become rate-limiting for the processing of the PPi product of the primary (polymerase) reaction. Specifically, the concentration of ATP sulfurylase was reduced from 300 milliUnits per assay reported elsewhere [23] to 0.03 milliUnits per assay, and at the same time the reaction volumes were also decreased four-fold to 25 µL. To demonstrate that under these reaction conditions the produced luminescence is directly proportional to the amount of PP_i_ present, we exogenously added 1–100 µM PP_i_ to luciferase, ATP sulfurylase and 100 µM adenosine 5′-phosphosulfate (APS) in the absence of RNA and 3Dpol. Indeed, luminescence measured within a spectral response range of 350 nm to 650 nm was directly proportional to PP_i_ concentration (Figure 1A).

To validate the assay we monitored the decrease in luminescence in presence of a positive control, 3′-deoxy-5-methyl uridine triphosphate (DMUT), which is a ribonucleoside analog that we predicted would block RNA synthesis because it lacks a 3′OH group (Figure 1B). Indeed, increasing amounts of DMUT suppressed the production of light (Figure 1B). The results from dose response experiments were plotted using Prism 4 (GraphPad Software Inc., CA) and an IC_50_ value of 0.68 µM was obtained for DMUT at midpoint concentrations. To ensure that DMUT does not interfere with the ATP sulfurylase and luciferase reactions we evaluated its effect on light produced by 1 µM of exogenously added PP_i,_ and were able to demonstrate that varying concentrations of DMUT has no effect on the production of light (data not shown). In order to assess the quality of luciferase based assay we also computed the Z-factor. Under our experimental conditions the Z-factor was 0.61 which is indicative of an excellent assay according to Zhang et al [24].

### Chemical Library Screening

**Figure 2:**
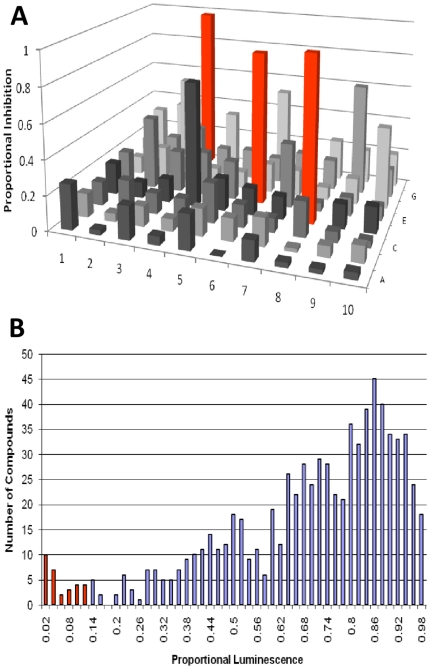
Relative inhibition of 3Dpol by compounds from a typical 96-well plate. (A) Relative inhibition of 3Dpol by compounds from a typical 96-well plate. 1.7 µM FMDV 3Dpol was incubated in the presence of 20 µM compounds, 25 mM Tris-HCl, pH 7.8, 40 nM poly-rA/5′-Cy3-dT_18_, 10 µM UTP, 25 mM KCl, and 1 mM MnCl_2_. After 60 minutes, ATP sulfurylase and luciferase assay components were introduced to the reactions as described in Materials and Methods. Luminescence was measured with a 96-well plate luminometer. Data are presented as percent luminescence inhibition (1 – [luminescence of a reaction/maximum luminescence] X 100). Red bars indicate compounds that inhibited by at least 88%. Compounds meeting this threshold were selected for further evaluation. (B) Frequency distribution of percent luminescence. Percent luminescence was calculated by dividing relative luminescence values of individual wells by the luminescence maximum of that plate.

**Figure 3:**
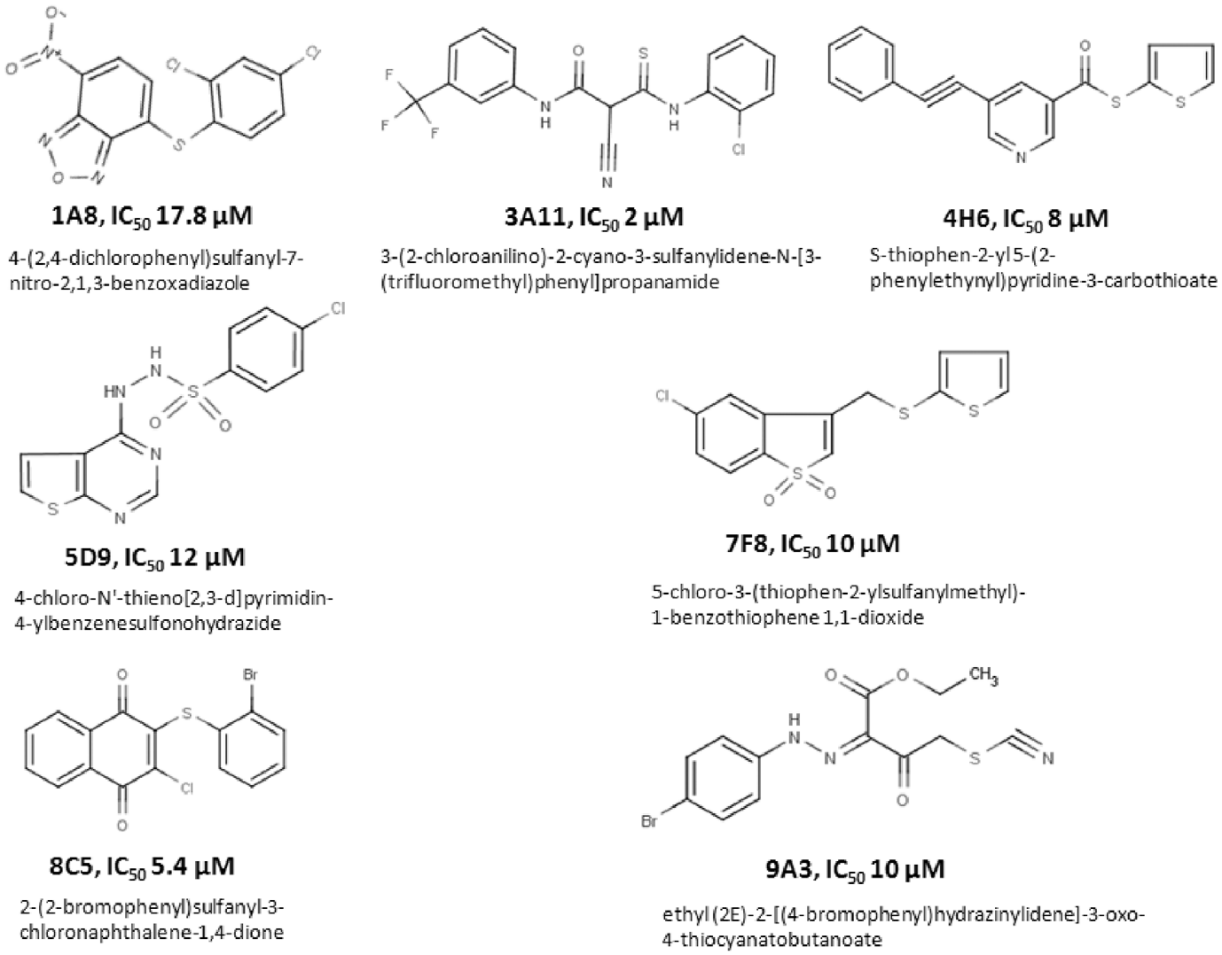
Chemical structures, names, and inhibition constants of 3Dpol inhibitors. The IC_50_ values of 3Dpol inhibition were determined by gel-based assays in at least four independent experiments, as described in the legend to Figure 4. Standard deviations were less than 5% in all cases.

**Figure 4:**
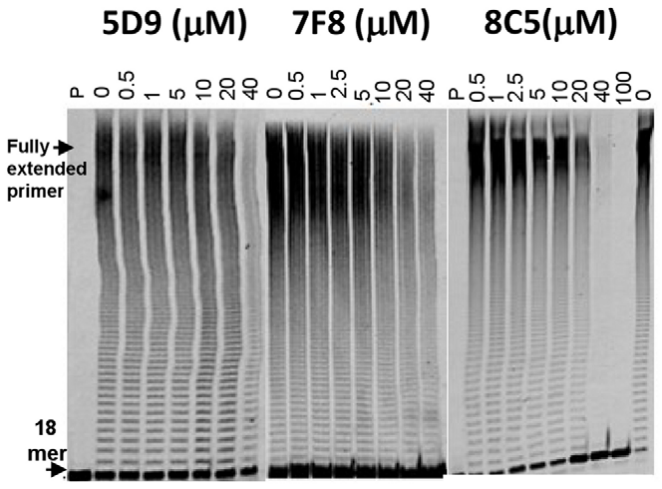
Gel-based validation of 3Dpol inhibition by different compounds. Representative results depicting the dose-dependent inhibition of RNA synthesis by 5D9, 7F8, and 8C5. RNA synthesis by 1 µM 3Dpol on 250 nM poly-rA/dT_18_ was carried out in the presence of varying concentrations of compounds (0–40 µM 5D9 and 7F8, and 0–100 µM 8C5) and 500 µM UTP in a buffer containing 50 mM Tris-HCl pH 7.8, 60 mM KCl, 0.01% BSA, 1 mM DTT and 0.1% NP40. Lanes labeled as P contain only free dT_18_ primer. To calculate the IC_50_, the amount of extended dT_18_ primer was plotted against the varying concentration of hits. The data points were fit to dose-response curves by GraphPad Prizm 4.0 (experiments were repeated at least 4 times).

Using this assay we screened ∼2,000 compounds from the Maybridge HitFinder library. Representative inhibition data from a single 96-well plate are shown in Figure 2. Typically, two to three compounds per plate suppressed FMDV 3Dpol activity by ∼90% (red bars in Figure 2A). Cumulative inhibition data are shown in Figure 2B. Based on these results, we selected 30 compounds that suppressed luminescence ≥90% (red bars in Figure 2B). These hits included compounds that inhibited the enzymatic activity of luciferase and/or ATP sulfurylase and consequently appeared as false positives. Therefore, to exclude false positives we validated the compounds' ability to specifically suppress 3Dpol activity with fluorescently labeled primer-extension assays. Approximately 2% of the tested compounds qualified as initial hits Most of these did not inhibit efficiently RNA synthesis by 3Dpol, as assessed by gel-based assays. Instead, they were shown to mostly interfere with the secondary luciferase assay reactions (data not shown). However, seven compounds (∼0.4% of compounds tested) did inhibit RNA synthesis by 3Dpol *in vitro* and were selected for further analysis. The chemical structures, the IC_50_ values, and the names of these compounds are shown in Figure 3. Two compounds, 1A8 and 3A11, had IC_50_s in the very low micromolar range (∼2 µM), whereas the other five (4H6, 7F8, 8C5, 9A3, 5D9) had values between 8 and 12 µM (Figures 3 and 4). Concentration-dependent analysis is shown in more detail for three of the compounds (Figure 4).

### Specificity of inhibitors

**Figure 5:**
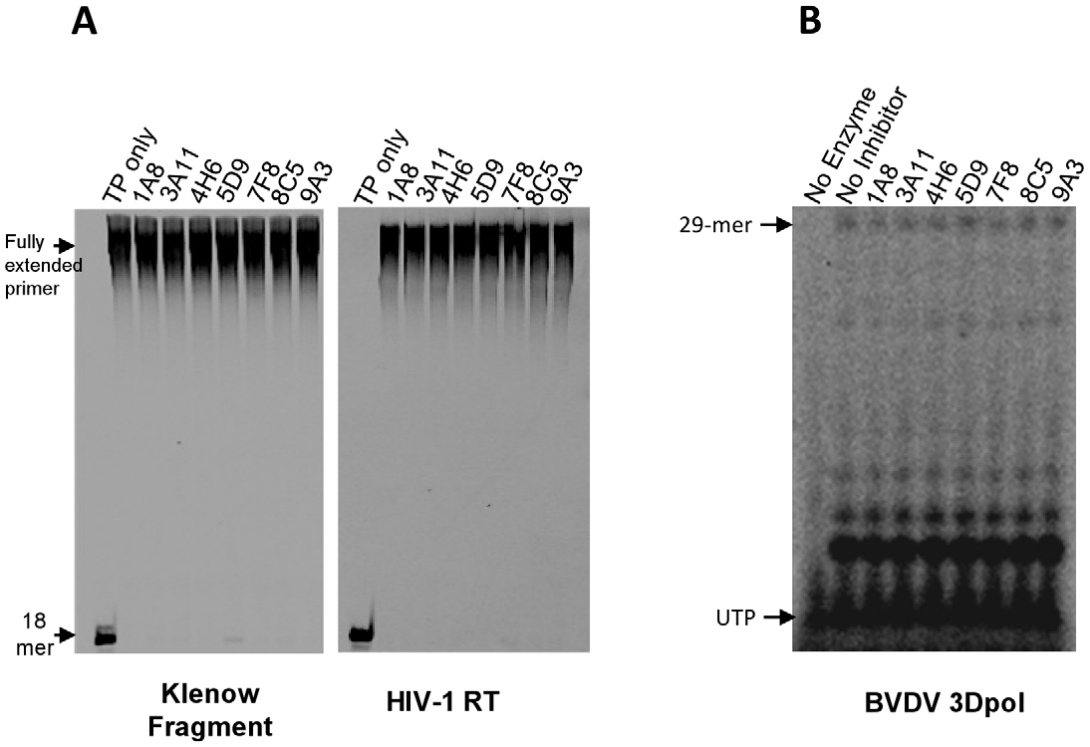
Effect of compounds on enzymatic activity of Klenow fragment and HIV reverse transcriptase. 10 nM KF or 20 nM HIV-1 RT were incubated with 250 nM poly-rA/dT_18_ and 20 µM of a 3Dpol inhibitor (1A8, 4H6, 6B11, 5D9, 7F8, 8C5, or 9A3) in a buffer containing 50 mM Tris-HCl, pH 7.8, 60 mM KCl, 1 mM DTT, 0.01% BSA, 0.1% NP40, and 4% DMSO. DNA synthesis was initiated by the addition of 1 mM MnCl2 and 500 µM dTTP final concentrations. No significant inhibition of KF or HIV RT is observed under these conditions. Panel B shows the RNA synthesis by BVDV 3Dpol in presence of 40 µM of each of the compounds. The RNA synthesis by BVDV 3Dpol was primed by GG dinucleotide in 50 mM Tris-HCl pH 7.8, pH 7.8, 60 mM KCl, 1 mM DTT, 0.01% BSA, 1 mM MnCl2, 100 µM ATP, GTP, CTP and 10 µM UTP mixed with 5 µCi of α-32P-UTP. The extension of GG can be seen as the radiolabeled bands where UTP is supposed to be incorporated.

The strong suppression of 3Dpol polymerase activity by the seven inhibitors could be due to non-specific binding of these inhibitors to poly-rA/5′-Cy3-dT_18_). To eliminate this possibility, we tested inhibition of polymerase activity by other unrelated enzymes that can use the same nucleic acid. Lack of inhibition under such conditions, would not only prove that the inhibitors do not block 3Dpol by binding to this T/P, but would also show that the inhibitors block specifically the 3Dpol target, without affecting the activity of other nucleic acid polymerases. Therefore, we tested the ability of the inhibitors to block nucleic acid synthesis by the DNA polymerase KF and the viral HIV-1 RT. Our results demonstrate that at concentrations comparable to those used for inhibition of 3Dpol (20 µM of inhibitor) the polymerase activity of these enzymes was clearly unaffected (Figure 5A). In addition, we monitored the effect of FMDV 3Dpol inhibitors on RNA synthesis by another RdRp, BVDV 3Dpol (Figure 5B). It is evident from the extension pattern of GG-dinucleotide that none of the compounds inhibited the RNA synthesis further supporting the idea that the inhibitors are specific for FMDV 3Dpol.

### Effect of inhibitors on the formation of 3Dpol-template-primer complex

**Figure 6:**
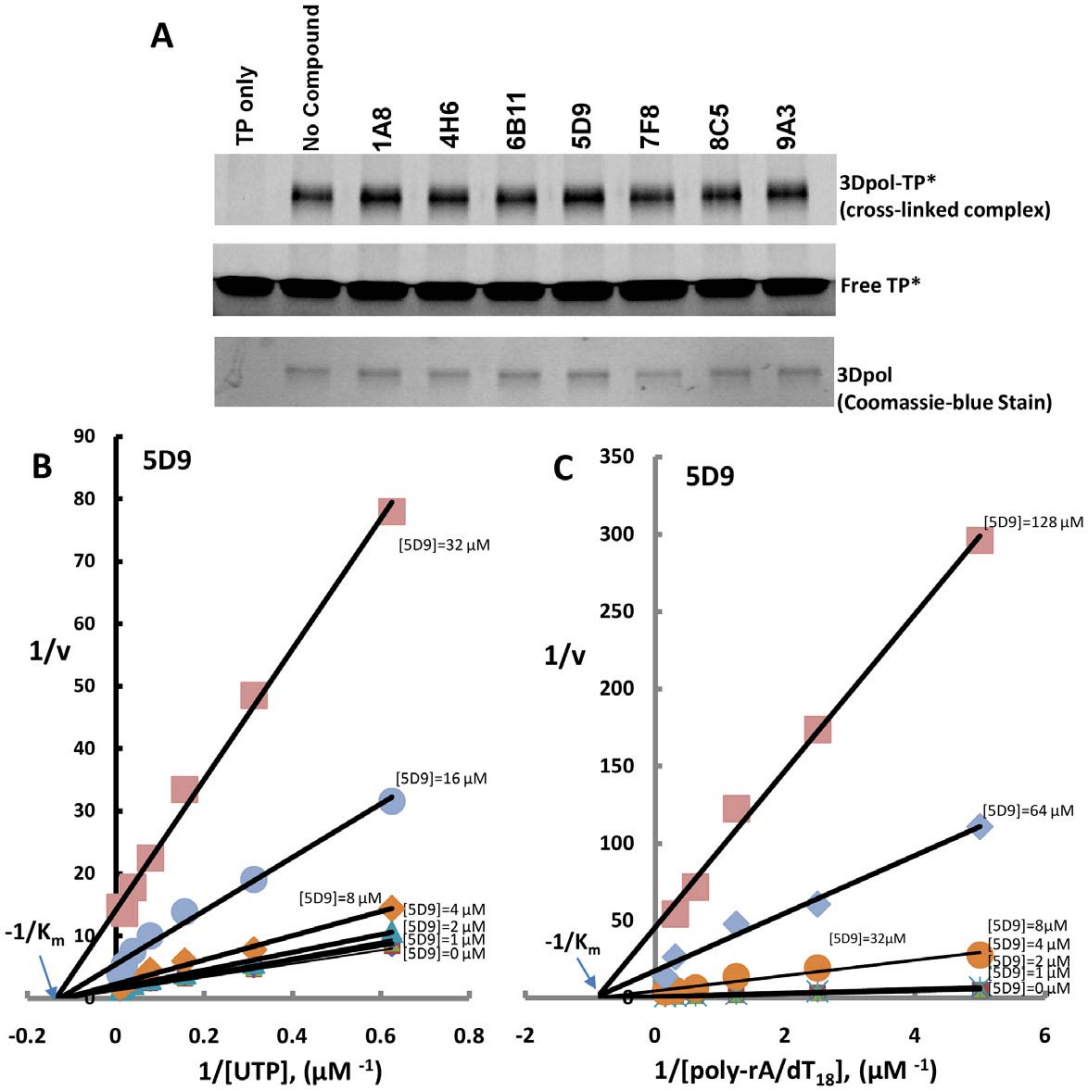
Inhibition of 3Dpol by inhibitors is non-competitive with respect to RNA and NTPs. (A) Effect of inhibitors on binding of 3Dpol to template-primer (T/P). Binding of 3Dpol to fluorescently labeled T/P (T/P*) was assessed by determining the amount of 3Dpol-T/P* covalent complex formed by UV-mediated cross-linking in the presence of 20 µM of inhibitors. For these experiments, 2 µg 3Dpol was incubated with 100 nM T/P (poly-rA/5′-Cy3-dT_18_) in a buffer containing 50 mM Tris-HCl pH 7.8, 1 mM DTT and 5 mM MgCl_2_ at 4°C for 10 minutes. Cross-linking was carried out by exposing the mixture to UV for 3 minutes as described in Materials and Methods. The radiograph at the top shows equivalent amounts of 3Dpol-T/P adduct in the absence and presence of the inhibitors. The middle and bottom figures in panel A show that the amounts of poly-rA/5′-Cy3-dT_18_ and protein, respectively are the same in all cross-linking experiments. (B) and (C) Non-competitive inhibition profile of FMDV 3Dpol by 5D9 under steady state conditions. Kinetic experiments with 3Dpol (1 µg/0.1 ml reaction volume) were conducted in 96-well plates using the luciferase-based assay (see methods) in the presence of increasing concentrations of 5D9 (0 to 32 µM), varying either UTP substrate (1.6-102.4 µM) (panel B) or poly-rA/dT_18_ (0.1 to 6.4 µM) (panel C). In both cases the X-axis intercepts (-1/K_m_ for the UTP or poly-rA/dT_18_ substrates) are not affected by the inhibitor concentrations, which is the hallmark of non-competitive inhibition. Indicated values are the means from at least three independent experiments.

In order to study the 3Dpol inhibition mechanism in more detail, we examined the possibility that the inhibitors affect the nucleic acid-binding step of the RNA polymerization reaction. Hence, we evaluated the formation of 3Dpol-T/P complex in the presence of inhibitory concentrations of these compounds. Binding was assessed by direct photo-chemical cross-linking of fluorescently labeled poly-rA/5′-Cy3-dT_18_ to 3Dpol. We have used this technique routinely to evaluate the effect of mutations or inhibitors on the ability of polymerases to bind nucleic acid [25], [26] [27]. The results of T/P cross-linking in presence and absence of the seven inhibitors (Figure 6, panel A) demonstrate that the presence of inhibitors does not significantly affect the amount of template-primer bound to 3Dpol. Thus, inhibition of 3Dpol is not mediated by interference with the binding of nucleic acid. Furthermore, these data provide physical evidence that the target site for the inhibitors is not in the T/P binding channel of 3Dpol.

### Kinetic mechanism of inhibition

We selected 5D9, the compound with the most potent antiviral activity (see below) for further kinetic characterization. We determined the kinetic mechanism of inhibition and K_i_ value. The Lineweaver–Burk plots in Figures 6B and 6C consist of a series of at least seven lines (one for each inhibitor concentration) intersecting at the same point on the X-axis (-1/K_m_) which is a hallmark of non-competitive inhibition [28], [29]. Hence, 3Dpol inhibition by 5D9 is non-competitive with respect to both the UTP and the nucleic acid substrates. The K_i_ value for 5D9 obtained from the Dixon plot (1/V *versus* [5D9], not shown) was 9.4±2.5 µM.

### Assessment of cytotoxicity

Inhibitors were evaluated for their effect on cell viability by the XTT cell viability assay. Uninfected cells were incubated in the presence of various doses of compounds (1, 5, 10, and 15 µM) for 24 and 48 hours. Little to no toxicity was demonstrated for all seven compounds at the indicated concentrations (data not shown). Cytotoxicity was also tested independently at higher concentrations of the compounds using the CytoTox-Glo assay (1, 10, and 100 µM of inhibitors) as described by the manufacturer (Promega, Madison, WI). Using this assay, we estimated that the 50% cytotoxic concentrations (CC_50_) of 5D9, 7F8, 4H6 and 9A3 were higher than 100 µM. Compounds 1A8, 8C5, and 3A11 were more cytotoxic with CC_50_s of 60, 55, and 70 µM, respectively.

### *In vivo* inhibition

**Figure 7:**
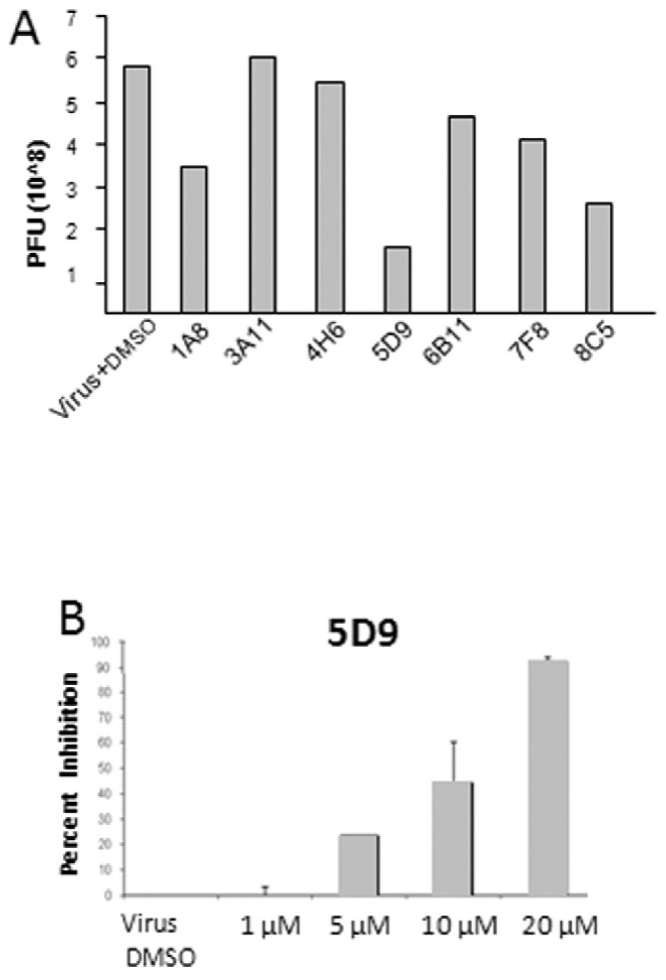
Inhibition of FMDV in cell-based assays. (A) Evaluation of anti-FMDV activity of 3Dpol inhibitors. Treatment with 15 µM of various compounds was performed following virus adsorption on BHK-21. The cells were further incubated in the presence of compound for another 24 hours and virus titer was determined by plaque assays (PFUs, plaque forming units) as described in the Materials and Methods. Experiments were done in duplicates. (B) Dose–dependence of FMDV inhibition by 5D9. Compound 5D9 was administered to BHK-21 cells in a dose-dependent manner prior to infection with FMDV. Post-infection, the compound was re-administered and incubated for another 24 hours. Following this incubation, samples were taken and plaque assays were performed. Results are reported as percent inhibition compared to a sample without inhibitor, and demonstrate a dose-dependent inhibition of virus replication. At the highest concentrations of 5D9 (20 µM) there was a greater than 90% inhibition of virus replication. Error bars represent standard error of the mean for two experiments. At these inhibitor concentrations no decrease in cell viability was observed.

We initially assessed the ability of the compounds to inhibit viral replication post-infection of BHK-21 cells with FMDV. The infected cells were incubated with various concentrations of the inhibitors and at specific time points. The effect on virus growth was assessed by plaque assay (Figure 7A and 7B). Early experiments with multiplicity of infection (MOI) of 0.1 did not show reproducible, significant inhibition of FMDV in the presence of inhibitors. At an MOI value of 0.01 we observed inhibition of viral replication by 15 µM of several compounds including 5D9, 8C5, and 1A8 (Figure 7A). In this study we focus on the most promising inhibitor, 5D9, because it exhibited the highest, dose-dependent, reproducible inhibition of FMDV at 24-hours after infection. Pre-treatment of BHK-21 cells with 5D9 prior to infection with FMDV A24 Cruzeiro and subsequent challenge with FMDV, followed by 24 hour growth in the presence of re-administered D59, resulted in suppression of viral growth in a dose-dependent manner and by as much as 90% at 20 µM concentration of D59 (Figure 7B). Notably, 5D9 did not exhibit significant cytotoxicity at these concentrations in parallel assays using the XTT method (data not shown).

### Identification of inhibitor binding site

**Figure 8:**
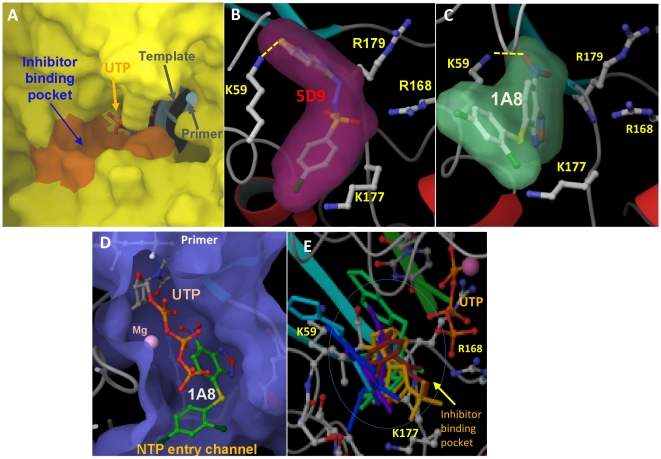
Poses of inhibitors docked at the inhibitor binding pocket of 3Dpol. Panel A shows a surface representation of the complex of 3Dpol (yellow) with RNA (gray ribbons for template and primer) and UTP (only the gamma phosphate of UTP is seen) (PDB code 2E9Z). The inhibitor binding site is shown in orange, proximal to the UTP binding site. Panels B and C show close-ups of the molecular surface areas of 5D9 (magenta) and 1A8 (green), respectively, docked at the inhibitor binding site of 3Dpol. Potential hydrogen bond interactions that involve the ε-NH_2_ of K59 and the inhibitors are indicated with yellow dotted lines. Some 3Dpol side chains have been removed for clarity. Panel D shows the position of the inhibitor binding pocket with respect to UTP with 1A8 docked at the inhibitor binding site. The potential NTP entry channel of 3Dpol is indicated with a yellow arrow. Atom types are displayed as: C, white; O, red; N, blue; S, yellow, P orange, and Cl, green. Panel E shows all inhibitors docked at the inhibitor binding pocket (1A8 in violet, 3A11 in blue, 4H6 in cyan, 5D9 in green, 7F8 in yellow, 8C5 in orange, and 9A3 in red), proximal to the UTP binding site.

Q-siteFinder identified 10 possible ligand-binding pockets. Almost all of these were smaller in size than the 3Dpol inhibitors, or were located within shallow surface crevices, very distant from the polymerase site. However, one potential ligand-binding pocket was in close proximity to the 3Dpol active site (Figure 8A). Docking of all seven compounds at this pocket was favorable and with significant glide scores. Interestingly, the binding pocket is pre-existing, and docking of the molecules required only small adjustments to the side chains of protein residues. Hence, using the ‘induced fit docking’ protocol, which permits more structural changes during the docking process, resulted in negligible changes in the binding mode of the inhibitors.

The inhibitor-binding pocket is proximal to, but not overlapping with the NTP binding site (Figures 8A). A close-up of the inhibitor-binding pocket with respect to the UTP-binding site and the possible NTP entry channel are shown in Figure 8D. The pocket is formed by residues V55, I56, S58, K59, R168, G176, K177, T178, R179 and I180. The energetically most favorable binding conformers of compounds 5D9 and 1A8 docked in FMDV 3Dpol are shown in Figures 8B and 8C, respectively. The seven 3Dpol-inhibitors docked at the inhibitor-binding site are shown in Figure 8E. The inhibitors appear to interact with residues of the binding pocket through both hydrophobic and electrostatic interactions. For example, the ε-NH_2_ group of K59 is shown to form hydrogen-bonds with the 5D9 and 1A8 inhibitors (Figure 8B and 8C). Similarly, residues K177 and R168 of 3Dpol are also within interacting distance with the inhibitors.

### Validation of the inhibitor binding pocket

**Figure 9:**
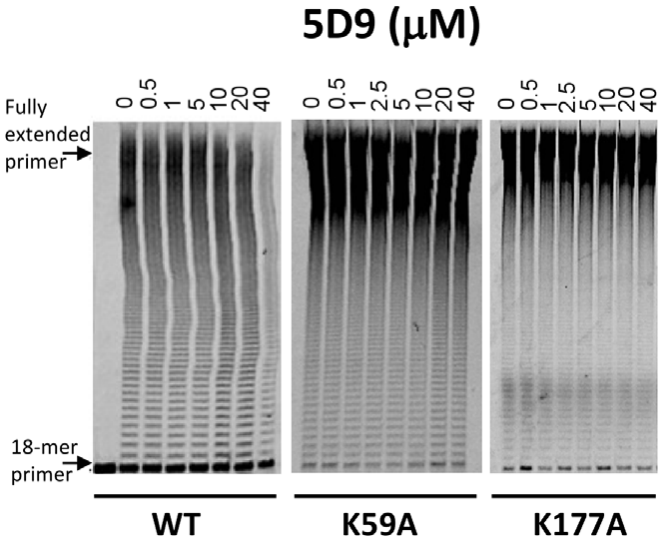
Susceptibility of WT and 3Dpol mutant enzymes to a 3Dpol inhibitor. Gel-based assays were used to evaluate the effect of K59A and K177A mutations on the susceptibility of 3Dpol to 5D9. Representative results depicting the dose-dependent inhibition of RNA synthesis by 3Dpol in presence of 5D9 are shown in this figure. RNA synthesis by 1 µM 3Dpol, 500 µM UTP, and 250 nM poly-rA/dT_18_ in a buffer containing 50 mM Tris-HCl pH 7.8, 60 mM KCl, 0.01% BSA, 1 mM DTT and 0.1% NP40 was carried out for 1 hour in the presence of varying concentrations of inhibitors (0–100 µM) and 500 µM UTP.

**Figure 10:**
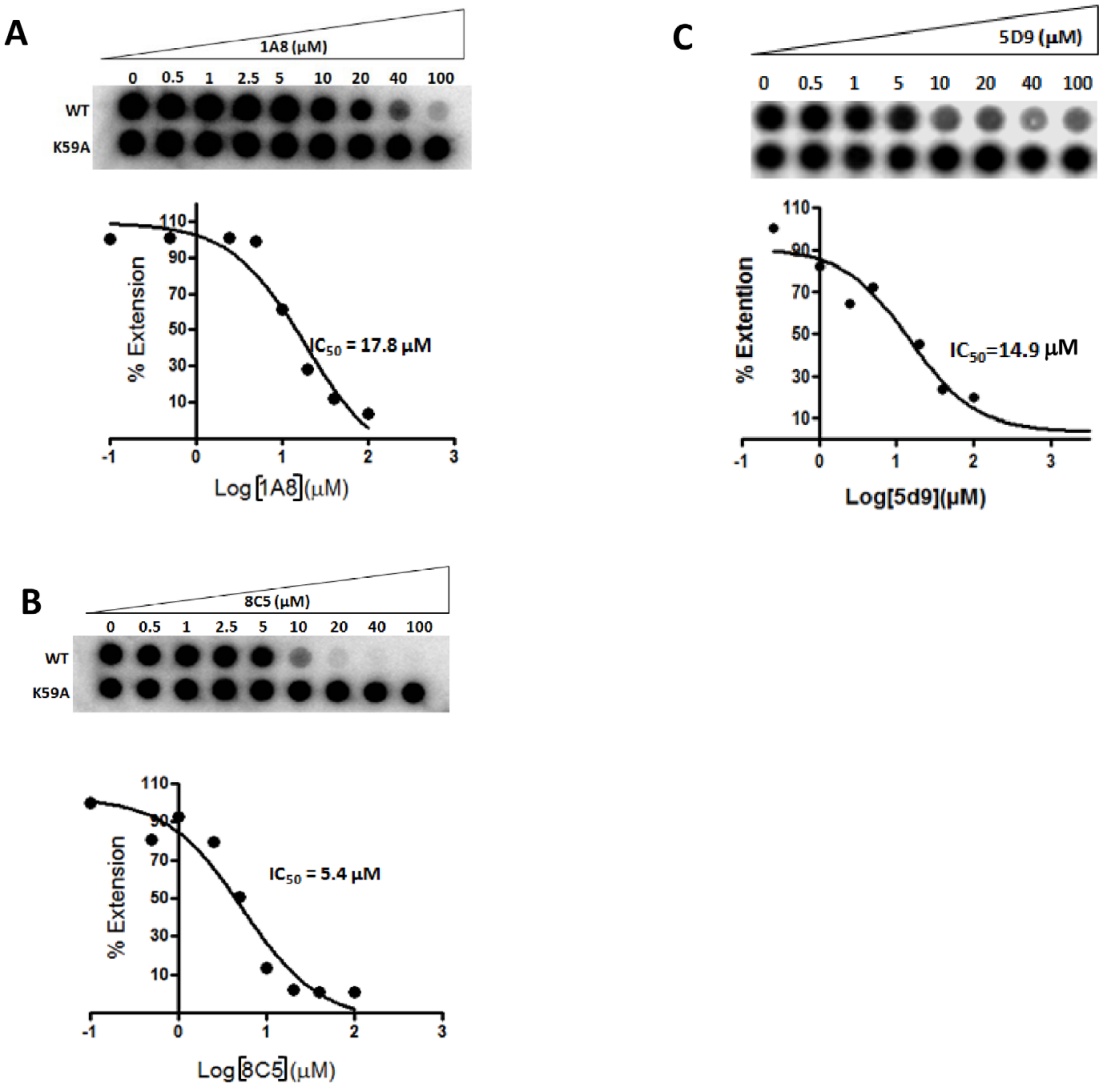
Filter binding assay to estimate the IC_50_ of 3Dpol inhibitors. 250 nM poly-rA/dT_18_ was incubated with 1 µM 3Dpol in 50 mM Tris-HCl pH 7.8, 60 mM KCl, 0.01% BSA, 1 mM DTT and 0.1% NP40 in presence of varying amounts of inhibitors (0–100 µM) (shown are representative results for 1A8, 8C5, and 5D9). Reactions were initiated by the addition of 1 mM MnCl_2_, 100 µM UTP (final concentration), and 0.125 µCi of α-^32^P-UTP per reaction. The reactions were allowed to proceed for one hour before quenching with 50 mM EDTA. Products were filtered through a charged nylon filter using a vacuum manifold apparatus (Whatman-GE Healthcare, Piscataway, NJ). The amount of radioactive material was quantitated by densitometery and plotted against the concentration of inhibitors. The data were fit to dose response curves using GraphPad Prism 4 to determine the IC_50_s.

In order to test the hypothesis that binding at this site blocks 3Dpol polymerase activity, we generated the K59A and K177A mutants of 3Dpol and tested their susceptibility to three inhibitors (5D9, 1A8, and 8C5). Under the conditions tested both mutants appeared to have polymerase activities comparable to the WT 3Dpol (Figures 9 and 10). However, results in Figures 9 clearly demonstrate that the site-directed 3Dpol mutants are resistant to 5D9. To better quantitate the inhibition of WT and mutant 3Dpols by the inhibitors we used a filter binding assay that can be run efficiently in a 96-well format. Results in Figure 10 demonstrate that WT is inhibited in a concentration-dependent manner and with IC_50_ values of 17.4, 5.4, 14,9 µM for 1A8, 8C5 and 5D9, respectively. These values are comparable to those determined by a gel-based assay (Figure 3). However, the activity of K59A 3Dpol remains unchanged even at the largest concentrations of 1A8 and 8C5 as well as 5D9, suggesting significant resistance to these inhibitors (Figure 10). Because the inhibitors were not readily soluble at concentrations significantly higher than 100 µM, we were not able to accurately determine the IC_50_ values of the mutant enzymes for these inhibitors. Nonetheless, the data in Figure 10 set lower limits for resistance of K59A to 1A8 and 8C5 (>5-fold and >20-fold, respectively).

## Discussion

There are currently no FDA-approved drugs for the treatment or prevention of FMD. Hence, control of the disease relies on slaughter of exposed animals and vaccination with chemically inactivated FMDV vaccines. However, these vaccines typically provide protection against one, or few of the 60 different FMDV subtypes. Moreover, they are unable to induce protection prior to 7 days post-vaccination. Thus, it remains important to continue the search for cost-effective compounds that inhibit most subtypes of FMDV. In this regard 3Dpol is a promising target because it has an important role in viral replication and its amino acid sequence is highly conserved among various serotypes. Moreover, FMDV 3Dpol has been studied extensively, both structurally and biochemically [30], [31], [32], [33]. These studies have provided important insights into the mechanism of RNA synthesis by FMDV 3Dpol and have established that this enzyme uses the same general catalytic mechanism as the polio virus RdRp [33], [34].

The synthesis of the minus strand RNA of FMDV is initiated by uridylylation of the small FMDV protein VPg, which is subsequently used by 3Dpol as a primer for continuing RNA synthesis in a template-dependent manner [30], [33]. Recent work has focused on the discovery of compounds that block the initiation or elongation steps of RNA synthesis. For example, Goris *et al*. [21] demonstrated that 2′-C-methylcytidine inhibits RNA synthesis of FMDV RNA [21]. Related analogs of this inhibitor have been reported to be incorporated into nascent RNA by the RdRp of HCV (NS5B), leading to chain termination [35]–[36]. Agudo *et al.* [14] recently demonstrated that 5-fluorouridine triphosphate, a nucleotide analog used in cancer therapy, has a dual (inhibitory and mutagenic) effect on FMDV RNA synthesis. This compound was also shown to compete with UTP for covalent binding to VPg. In a recent study, Furata *et al*. have proposed that nucleoside analog T1106 suppresses FMDV replication through inhibition of FMDV RdRp by an unknown mechanism [22]. Another study demonstrated that RNA aptamers specific to 3Dpol inhibit efficiently the RdRp activity of the enzyme *in vitro* [37]. However, aptamers are highly charged oligonucleotide molecules that may be limited by low bioavailability, and difficulty to deliver to intracellular targets. Finally, ribavirin, a mutagenic nucleotide analog that has been approved for the treatment of HCV infections, has also been reported to have antiviral activity against FMDV [13]. However, a single residue mutation in 3Dpol (M296I) resulted in decreased capacity of the FMDV enzyme to use ribavirin triphosphate as a substrate in the place of GTP and ATP [19]. These examples illustrate the increasing importance and challenges of targeting 3Dpol of FMDV.

In this study we screened a chemical library of small molecules for inhibitory activity against 3Dpol. This chemical library contains compounds that follow Lipinski's rule of five, an empirical rule that is used to evaluate “drug-likeness” and potential for oral bioavailability in humans [38]. To identify potential inhibitors, we used a non-radioactive assay to monitor the RdRp activity of 3Dpol by measuring the release of pyrophosphate (PP_i_) generated during synthesis of the nascent RNA strand [23]. The coupled-enzyme assay used ATP sulfurylase and firefly luciferase enzymes to generate luminescence, proportional to the concentration of pyrophosphate. This assay enabled initial selection of compounds that suppressed luminescence. Compound hits were validated in a primer extension assay (Figure 4). Some of the initial hits were false positives, as they turned out to be inhibitors of either ATP sulfurylase and/or firefly luciferase. This was confirmed by their ability to suppress luminescence generated by exogenously added PP_i_ rather than from PP_i_ generated from RdRp reaction. One early concern was that the inhibitors might block polymerases non-specifically, by binding to the minor groove of the nucleic acid substrate. This possibility was eliminated by demonstrating that the inhibitors did not affect the polymerase activity of two unrelated polymerases, HIV RT and KF, using the same nucleic acid as substrate (poly-rA/dT_18_).

Seven 3Dpol inhibitors were tested at the Plum Island Animal Disease Center for *in vivo* (cell culture) FMDV inhibitory effects. Their antiviral properties were evaluated using FMDV subtype A24 Cruzeiro. Given the high homology of FMDV 3Dpol among different serotypes it is likely that the compounds will have antiviral activity for other FMDV serotypes as well. The cell-based assays indicated that 5D9 had the most pronounced and consistent antiviral effect (EC_50_  = 12 µM, EC_9_  = 20 µM). Further cytotoxicity measurements indicated that the CC_50_ for 5D9 was higher than 100 µM. Since the antiviral activity of the compound is 12 µM, the therapeutic index (CC_50_/EC_50_) is larger than 8. We are currently evaluating analogs of the original hits to identify compounds with improved antiviral properties. Nonetheless, the antiviral potency of 5D9 is ∼80 fold better than that of ribavirin (EC_50_ = 970 µM, EC_90_ = 1697 µM), and comparable to 2′-*C*-methylcytidine (EC_50_ = 10 µM, EC_90_ = 15 µM) [21].

5D9 is a good candidate for use in combination with compounds that inhibit the virus through different strategies, including peptide-conjugated morpholino oligomers that target the 5′ and 3′ untranslated regions (UTRs) of the FMDV genome [31].

In addition to identifying novel FMDV inhibitors, we also demonstrated that their mechanism of action involves targeting a previously unidentified inhibitor binding pocket. We took advantage of the multiple crystal structures of FMDV 3Dpol that have been solved by the Verdaguer and Domingo groups [20], [32], [39], [40]. These structures represent various reaction intermediates and provide key information on understanding the molecular details of RNA-dependent RNA polymerization. We used molecular modeling tools to search for possible binding sites in the 3Dpol structures. We identified a site where all compounds could be docked efficiently (Figure 8E). The binding site is pre-existing, and efficient docking of the compounds does not require significant conformational changes in side-chain or main-chain atoms. Moreover, the binding pocket is highly conserved in several crystal structures of various complexes, including: 1) the 3Dpol structure containing template-primer, incoming UTP and released PPi (PDB code 2E9Z), 2) the structure of unliganded 3Dpol (PDB code 1U09), 3) the structure of RNA-bound binary complex (PDB code 1WNE), 4) uridylylated VPg-bound complex (PDB code 2F8E), 5) the structure of 3Dpol complexed with RNA template-primer and ATP (PDB file 2EC0), 6) 3Dpol complexed with RNA template-primer, ATP and UTP (PDB code 2E9Z), 7) 3Dpol complexed with template-primer RNA and 5F-UTP (PDB code 2E9T) and 8) 3Dpol complexed with template-primer RNA and ribavirin (PDB code 2E9R). Hence, the molecular modeling studies predicted that the inhibitors can bind to multiple intermediates of the enzymatic reaction, as expected from non-competitive inhibitors. This prediction is consistent with our experimental results that demonstrate that the mode of inhibition by 5D9 and 7F8 is indeed non-competitive. Detailed analysis of the mechanism of inhibition and resistance of all seven inhibitors will be published elsewhere. Importantly, residues that line the inhibitor-binding pocket are entirely conserved among all 60 FMDV subtypes. Hence, we expect that unlike vaccines that are subtype-specific, small molecule inhibitors that target this pocket can have broad antiviral properties.

The inhibitor binding pocket is proximal to, but distinct from, the NTP binding site of 3Dpol (Figures 8A and 8D). The compounds are expected to bind in a channel that may be used for entry of the nucleotide substrates and exit of the PPi product (Figure 8D) [32]. Interestingly, the non-competitive type of inhibition is reminiscent of the mechanism by which non-nucleoside reverse transcriptase inhibitors (NNRTIs) block the DNA polymerase activity of HIV RT [41], [11], [42], [43], or other non-nucleoside inhibitors that inhibit the RNA polymerase NS5B of Hepatitis C Virus [44] [28] [45], [46], [47], [48]. The molecular details of the inhibition and resistance mechanism will be elucidated by ongoing pre-steady state kinetic and crystallographic studies. Such studies should also facilitate structure-based drug design of more potent antivirals that target 3Dpol. The highly conserved inhibitor-binding pocket will provide numerous opportunities for hydrophobic interactions between the aromatic/hydrophobic ring systems of the inhibitors and the hydrophobic residues the line the pocket (V55, I56, and I180, or the aliphatic chains of residues K59, K177, R168, R179) or hydrogen-bond or hydrophilic interactions between the inhibitors and the ε-NH_2_ of K59, K177, or the guanidinium groups of R168 and R179.

It is noteworthy that two of the seven inhibitors, 5D9 and 7F8, contain sulfonamide or sulfone chemical groups. Similar compounds have long been used for the treatment of bacterial, viral and other infections [49]. Hence, we are currently performing structure-activity studies to improve the potency of these inhibitors.

In conclusion, our study demonstrates that we have identified a pocket in 3Dpol that can be targeted by inhibitors that block RNA synthesis through a non-competitive inhibition mechanism. These compounds can have antiviral properties and could be used for blocking FMDV replication. Further studies may lead to the development of even more potent small molecule inhibitors of FMDV that can be used as vaccine-alternatives and/or offer additional options for the prevention and containment of FMDV during outbreaks.

## Materials and Methods

### Materials

The Maybridge-Hitfinder chemical library of compounds (version 6) was purchased from Maybridge, (Thermo Fisher Scientific, Cornwall, United Kingdom). Screening reactions were carried out in Microfluor 2 black U-bottom 96-well plates (Fisher Scientific). Oligonucleotides were purchased from Fermentas, (Glen Burnie, Maryland). Ultrapure nucleotides were purchased from Novagen (Madison, Wisconsin) and 3′-deoxy 5-methyl-uridine 5′-triphosphate (DMUT) was obtained from TriLink BioTech (San Diego, CA). Compound hits were also purchased independently from Ryan Scientific Inc. (Mt. Pleasant, SC) for independent validation of the inhibition results.

### Expression and purification of WT, K59A and K177A FMDV 3Dpol

Plasmid pET-28a containing the FMDV 3Dpol coding sequence with an AAALE linker at the carboxyl terminus followed by 6 histidines was obtained from Drs. Verdaguer and Domingo [32]. It was transformed into the Rosetta 2 expression strain (Novagen). Kanamycin-resistant colonies were grown at 37°C and induced at A_600_ of 0.9–1.0 by the addition of 1 mM isopropyl β-D-1-thiogalactopyranoside (IPTG). The cells were harvested by centrifugation (4,500 *g*, 20 min) and stored at -20°C. Frozen cell pellets were resuspended in buffer A (25 mM Tris-HCl pH 8.0, 500 mM NaCl and 5% glycerol). The protein was purified by nickel-affinity chromatography with a gradient of 25 mM to 500 mM imidazole in buffer A. Fractions containing pure protein (∼95%) were pooled and dialyzed against the storage buffer containing 12.5 mM Tris-HCl pH 8.0, 100 mM NaCl and 50% glycerol. The protein concentration was determined using a Nanodrop spectrophotometer (Thermo Scientific, Wilmington, DE) and confirmed by comparison to known amounts of coomassie-stained Bovine Serum Albumin.

The K59A and K177A mutants were prepared using the Stratagene QuikChange site-directed mutagenesis kit as described by the manufacturer, and expressed and purified as described above for the WT enzyme.

### 96-well plate screening assay

The Maybridge-Hitfinder library of compounds was screened using a luciferase-based assay similar to the one developed by Lahser and Malcolm [23], which quantitates the pyrophosphate (PP_i_) product of nucleic acid synthesis. The chemical library was supplied as lyophilized films in a 96-well plate format. We used a Precision Microplate Pipetting system (Winooski, VT) to suspend the compounds in 100% dimethyl sulfoxide (DMSO) to a final concentration of 10 mM (‘mother plates’). From these plates several sets of ‘daughter plates’ containing 500 µM of each compound (in 100% DMSO) were generated and stored at −80°C.

The polymerase reactions that generate pyrophosphate product were carried out in a 96-well format. Specifically, each well contained 25 µl of reaction mixture containing 25 mM Tris-HCl, pH 7.8, 25 mM KCl, 1.7 µM 3Dpol, 10 µM UTP, 1 mM MnCl_2_ and 20 µM inhibitor. The polymerase reaction was initiated by the addition of 40 nM of poly-rA/dT_18_. The reactions were allowed to proceed for 1 hour at 37°C followed by 10 minutes incubation on ice. The released pyrophosphate (PPi) from the polymerase reaction was subsequently quantitated by adding to the above polymerase reaction mixture a luciferase reaction mixture (25 µl) containing 4.8 nM luciferase, 6×10^−5^ units of adenosine-5′-triphosphate sulfurylase (ATP_sulfurylase_) (Sigma Aldrich, St. Louis, MO), 5 µM adenosine-5′-phosphosulfate (APS), 310 µM d-luciferin, 0.5 mM coenzyme-A, 25 mM Tris-HCl pH 7.5, and 50 mM NaCl. Luminescence was measured immediately with a Veritas microplate luminometer (Turner BioSystems Sunnyvale, CA).

### Gel-based primer extension assay for validation of inhibitors

Compounds that suppressed production of light during the screening of the library were validated directly by measuring their ability to inhibit the RNA-dependent RNA polymerase activity of 3Dpol in gel-based primer extension assay, as we have done previously [50], and using optimized conditions stated below. All concentrations represent the final concentrations in the reactions unless otherwise indicated. The RNA synthesis by 1 µM 3Dpol using 250 nM fluorescently labeled poly-rA/5′-Cy3-dT_18_ was carried out in the presence of varying concentrations of inhibitors (0–40 µM or 0–100 µM) and 500 µM UTP in a buffer containing 50 mM Tris-HCl pH 7.8, 60 mM KCl, 0.01% BSA, 1 mM DTT and 0.1% NP40. The compounds were dissolved in DMSO, and the final concentration of DMSO in the reactions (including the controls) was 4%. Reactions were initiated by the addition of MnCl_2_ to a final concentration of 1 mM, and allowed to proceed for 1 hour at 37°C before quenching with 95% formamide. Inhibition of RNA polymerization was monitored by resolving the primer extension products on 16% polyacrylamide-8 M urea gels, followed by scanning of gels on a Fuji FLA-5000 Fluorometer. Bands corresponding to full extension products were quantified using the Fujifilm MultiGauge software (Stamford, CT). IC_50_s were obtained from dose-response curves using GraphPad Prism 4. All inhibition experiments were performed independently at least three times.

### Filter binding assay for assessment of the susceptibility of the K59A or K177A 3Dpol mutants to the inhibitors

The effect of mutations on the susceptibility of 3Dpol to various compounds was assessed using two assays. First, by a gel-based primer extension assay described above, and second, by a 96-well plate filter binding assay. The latter assay allowed fast and accurate calculation of IC_50_s by measuring the incorporation of ^32^P-UMP by the WT and mutant 3Dpols into poly-rA/dT_18_ template-primer (T/P), in the presence and absence of inhibitors. Assays were carried out in a final volume of 20 µl containing 1 µM enzyme and 50 mM Tris-Cl pH 7.8, 1 mM DTT, 0.01% BSA, 0.1% NP40, 60 mM KCl, 250 nM unlabeled poly-rA/dT_18_ and varying concentrations (0–100 µM) of inhibitors. Reactions were initiated by the addition of 100 µM UTP, α-^32^P-labeled UTP (0.5 µCi/nmol) and 1 mM MnCl_2_ (final concentrations). After incubation at room temperature for 1 hour, the reactions were terminated by the addition of 50 mM EDTA. The reaction products were then passed through a positively charged nylon membrane using a vacuum manifold apparatus (Whatman-GE Healthcare, Piscataway, NJ). Extended primers that had been radiolabeled by the incorporation of α-^32^P-UMP were bound to the filter, whereas the unincorporated α-^32^P-UTP was filtered through the membrane. Radioactive filters were exposed to phosphor screens followed by phosphorimaging and analyzed using the Multi Gauge V3.0 software (FujiFilm). Dose response curves were plotted using GraphPad Prism 4 to determine the IC_50_s.

### Specificity of inhibitors for FMDV 3Dpol

To establish that the compounds did not inhibit the 3Dpol activity by merely chelating with the nucleic acid, we monitored the effect of 20 µM of the inhibitors on the DNA synthesis by two unrelated nucleic acid polymerases, using the same template-primer system (poly-rA/dT_18_). Hence, we measured DNA synthesis by 10 nM of the Klenow Fragment (KF) of *E. coli* DNA polymerase I or 20 nM of the HIV-1 reverse transcriptase (HIV-1 RT), using 250 nM fluorescently labeled poly-rA/5′-Cy3-dT_18_ (37°C for 1 hour), in 50 mM Tris-HCl, pH 7.8, 60 mM KCl, 1 mM DTT, 0.01% BSA, 1 mM MnCl2 and 500 µM dTTP. All reactions, including controls or in the presence of inhibitors, contained 4% DMSO.

In addition to assessing the DNA synthesis by KF and HIV-1 RT, we also tested the ability of compounds to inhibit the RNA synthesis by Bovine Virus Diarrhea Virus RNA polymerase (NS5B). The RNA synthesis in absence and presence of 40 µM compounds was carried out in a buffer containing 50 mM Tris-HCl pH 7.8, pH 7.8, 60 mM KCl, 1 mM DTT, 0.01% BSA, 1 mM MnCl2, 100 µM ATP, GTP, CTP and 10 µM UTP mixed with 5 µCi of α-32P-UTP. The incorporation by 1 µM NS5B was monitored on 1 µM 31-mer template **(**5′-CCAUAGAUAGCAUUGGUGCUCGAACAGUGAC-3′) using GG as the priming sequence.

### Effect of inhibitors on the ability of 3Dpol to bind nucleic acid

To assess if the inhibitors interfere with the binding of nucleic acid by 3dpol, we used UV-mediated cross-linking of template-primer to 3Dpol in the presence of the inhibitors. For UV-mediated cross-linking, 2 µg enzyme were incubated with 100 nM of fluorescently labeled poly-rA/5′-Cy3-dT_18_ in a buffer containing 50 mM Tris-HCl pH 7.8, 1 mM DTT and 5 mM MgCl_2_ at 4°C, for 10 minutes. The samples were exposed to UV light (254 nm) at a dose rate of 125 mJ/cm^2^ for 3 min using a BioRad GS Gene Linker UV chamber (BioRad Laboratories, CA), as described previously [25], [26] [27]. Measurement of covalent attachment of labeled poly-rA/5′-Cy3-dT_18_ to enzyme was assessed by 8% SDS-polyacrylamide gel electrophoresis. Bands corresponding to 3Dpol cross-linked to template-primer were visualized by a Fuji FLA-5000 phosphorimager and quantified using FujiFilm MultiGauge (Stamford, CT).

### Enzyme kinetics

To determine the inhibition mode with respect to UTP (competitive or non-competitive), we used the 96-well luciferase–based assay to measure the effect of 5D9 in the RdRp activity of 3Dpol. 25 µl reactions containing 200 nM 3Dpol, 25 mM Tris-HCl, pH 7.8, 50 mM KCl, 1 mM MnCl_2_ and varying 5D9 (0–32 µM) were initiated by adding 4 µM poly-rA/dT_18_. These reactions also included increasing amounts of UTP (1.6–102.4 µM). For determining the inhibition mode with respect to nucleic acid, the poly-rA/dT_18_ concentration was varied (from 0.1 to 6.4 µM) in the presence of 50 µM UTP and increasing amounts of 5D9 (from 0–128 µM). The reactions were allowed to proceed for 30 minutes at 37°C, followed by 10 minutes incubation on ice. Released PPi was quantitated as described above for the 96-well plate screening assay. Assays were carried out in three independent experiments. Results were analyzed in Lineweaver–Burk graphs (1/V *vs.* 1/[*S*] for various inhibitor concentrations) using GraphPad Prism. Dixon plots (1/V *vs.* [5D9]) were used to determine the inhibitor K_i_ from the X-axis intercept [51].

### Assessment of cytotoxicity

We assessed the effect of the inhibitors on cellular viability using a commercially available kit (Roche Diagnostics, Indianapolis, IN) that measures metabolization of XTT 2,3-bis(2-methoxy-4-nitro-5-sulfophenyl)-5-[(phenylamino) carbonyl]-2H-tetrazolium hydroxide). XTT is a tetrazolium salt that is reduced to a soluble orange-colored formazan product by mitochondrial succinate dehydrogenase, which retains activity in metabolically active cells. The amount of this product is proportional to the number of living cells and can be spectrophotometrically quantified. The assay was performed in triplicates as follows: Baby hamster kidney (BHK-21) cells **(**American Type Culture Collection, Manassas, VA) were seeded to 90% confluency in a 96-well plate. Cells were incubated for 24 hours with 1, 5, 10, and 20 µM of inhibitors in a final volume of 100 µL phosphate buffered saline (PBS). Cells treated with Triton X-100 were used as a control for loss of cell viability. After the 24-hour incubation period the PBS was removed and replaced with phenol red-free media. 20 µL of XTT was added per well and cells were incubated for an additional 3.5 hours. After incubation, the optical density at 450 nm was read by a plate reader. We also assessed the cytotoxic effect of the inhibitors independently using the CytoTox-Glo kit (Promega, Wisconsin), which measures cleavage of a luminogenic AAF-Glo substrate by dead-cell proteases. Assays were carried out as described by the manufacturer [52]. Following cleavage, a substrate for luciferase (aminoluciferin) was released, resulting in the luciferase-mediated production of light. Percent of dead cells were determined in the presence of inhibitor concentrations up to 100 µM.

### *In vivo* assessment of inhibition

For the initial assessment of the antiviral compounds (Figure 7A), BHK-21 cells were grown in BME BHK-21 medium supplemented with 10% bovine calf serum. BHK-21 cells were seeded (150,000 per well) in a 24-well plate in antibiotic-free media. They were subsequently infected with 0.01 multiplicity of infection (MOI) of FMDV A24 Cruzeiro and incubated for1 hr at 37°C to allow for virus adsorption. Subsequently, the cells were treated with ice-cold 2-morpholinoethanesulfonic acid (MES)-buffered saline (25 mM MES pH 5.5, 145 mM NaCl) to inactivate residual virus particles and then rinsed twice with minimal essential media (MEM) (Invitrogen, Carlsbad, CA) containing 1% fetal bovine serum (FBS) and 25 mM HEPES (pH 7.4). Inhibitors were added to the infected cells and cells were further incubated at 37°C for 24 hours. At this time, plates were frozen for subsequent determination of virus titers. These were determined by plaque assays using a 1% gum tragacanth overlay, and the mixture was incubated for 24 h at 37°C. The plates were fixed and stained for analysis with crystal violet, 0.3% in HistoChoice (AMRESCO, Solon, OH), and the plaques were counted as described previously [31].

The experiments on characterization of 5D9 (Figure 7B) were carried out as follows: 5D9 was added in a dose-dependent manner to BHK-21 cells and incubated for one hour to allow for absorption of the inhibitor. Cells were washed prior to infection to remove any residual DMSO, which could potentially modify virus adsorption. Subsequently, cells were infected by adding FMDV A24 Cruzeiro (MOI of 0.01) and incubated for 1 hour at 37°C to allow for virus adsorption. After washing and neutralizing the cells as described above, 5D9 was re-administered in a dose-dependent manner and the infected cells were further incubated with the inhibitor for 24 hours at 37°C. At this time, plates were frozen and plaque analysis was carried out as described above. Assays were performed in triplicate and included controls with DMSO (0.5% in media) and no inhibitor.

### Search for potential inhibitor binding sites

We used the crystal structure coordinates of the FMDV 3Dpol complex with RNA, UTP and PPi (PDB file 2E9Z) to search for potential binding sites of the inhibitors, using the Q-siteFinder program [53]. The potential inhibitor binding sites were initially evaluated by size (if they were large enough to allow docking of the inhibitors. For those sites that were of the right size we performed additional docking studies of the various inhibitors. For this purpose we generated molecular models of the compounds based on structure data files (sdf) using LigPrep, a ligand preparation tool that is interfaced with Maestro (Schrodinger Inc. NY). The structures generated by LigPrep were docked into the ternary complex of FMDV 3Dpol with RNA and UTP (PDB file 2E9Z) using the software ‘Glide’ with extra precision (XP) and ‘Induced Fit Docking’ workflow incorporated in Maestro (Schrodinger Inc. NY).
